# Do we need new prokinetics to reduce enteral feeding intolerance during critical illness?

**DOI:** 10.1186/s13054-016-1466-3

**Published:** 2016-09-24

**Authors:** Arthur Raymond Hubert van Zanten

**Affiliations:** Department of Intensive Care Medicine, Gelderse Vallei Hospital, Willy Brandtlaan 10, 6716 RP Ede, The Netherlands

**Keywords:** Promotility agents, Prokinetics, Feeding intolerance, Gastric emptying, Gastric residual volume, Metoclopramide, Erythromycin, Ghrelin

## Abstract

Gastrointestinal feeding intolerance and critical illness-associated gastric motility dysfunction are common. Although recent guidelines recommend not interrupting gastric feeding when gastric residual volume (GRV) is lower than 500 mL or to completely abandon measurement of GRV, it may seem that the relevance of prokinetics is reduced.

In patients at risk for aspiration and in multimodal strategies to enhance feeding performance, however, use of prokinetics is still advocated. Metoclopramide and erythromycin are commonly used promotility agents, although with relevant side effects.

Potential targets for new agents and early study results are addressed.

## Commentary

A clear consensus definition on acute gastrointestinal injury during critical illness is lacking. Gastrointestinal feeding intolerance (GFI) is usually defined by vomiting, abdominal distention, complaints of discomfort, high nasogastric tube output, high gastric residual volumes (GRVs) measured at intervals, diarrhea, reduced passage of flatus and stool, or abnormal abdominal radiographs [1]. In daily practice, ICU nurses typically assess intolerance solely by measuring GRVs, with common threshold levels for interrupting enteral nutrition (EN) ranging from 200–250 mL [2]. GFI may be encountered in 50 % of ventilated patients, depending on the diagnosis, premorbid condition, ventilation mode, medications, and metabolic state [3].

The recently updated guidelines for adult critically ill patients by the Society of Critical Care Medicine (SCCM) and American Society for Parenteral and Enteral Nutrition (ASPEN) suggest that, in most patients, it is acceptable to initiate EN in the stomach. Patients should be monitored daily for tolerance of EN and inappropriate cessation of EN should be avoided. Administration should be diverted lower in the gastrointestinal tract (post-pyloric feeding) in patients at high risk for aspiration or in case of gastric EN intolerance [4].

Now here a clinical dilemma may arise: The most likely routine to monitor GFI is to measure GRV. However, GRVs do not correlate with incidences of pneumonia, regurgitation, or aspiration. GRVs were shown to have very low sensitivity and positive and negative predictive value to predict aspiration [4]. Raising the cutoff value for GRVs (to interrupt EN administration) from 50–150 to 250–500 mL does not increase the incidence of complications. Moreover, recent studies have suggested that it is safe to completely abandon measuring GRV and as a consequence nutritional targets are better achieved, although more vomiting was noticed in one study [5, 6].

Combining evidence and pragmatism ASPEN/SCCM recommends GRVs not be used as part of routine care to monitor ICU patients receiving EN. However, for those ICUs where GRVs are still utilized, holding EN for GRVs <500 mL in the absence of other signs of intolerance should be avoided [4].

Should we then also abandon use of prokinetics to improve feeding performance in case gastric emptying is reduced as reflected by increased GRVs? Probably not.

For patients at risk for aspiration it is recommended to initiate promotility agents, such as metoclopramide and erythromycin, where clinically feasible [4]. However, risk factors are present in almost all ICU patients [7]. Moreover protocols applying volume-based feeding in conjunction with prokinetics from the initiation of feeding and that only stop administration of these promotility agents if no longer required have been shown to increase EN delivery while reducing the incidence of nosocomial infections compared with ICUs without such protocols [8, 9].

The most commonly used prokinetics are metoclopramide and erythromycin. Erythromycin use has been associated with cardiac toxicity, tachyphylaxis, and bacterial resistance [10]. Adverse complications of metoclopramide include tardive dyskinesia, akathisia related to the rate of infusion, and various cardiovascular side effects, although reported adverse events in critically ill patients are rare [11].

Both agents may confer QT prolongation, predisposing to cardiac arrhythmias. Another problem of prokinetics is the occurrence of tachyphylaxis, the phenomenon that treatments become less effective during prolonged administration [11].

**Fig. 1:**
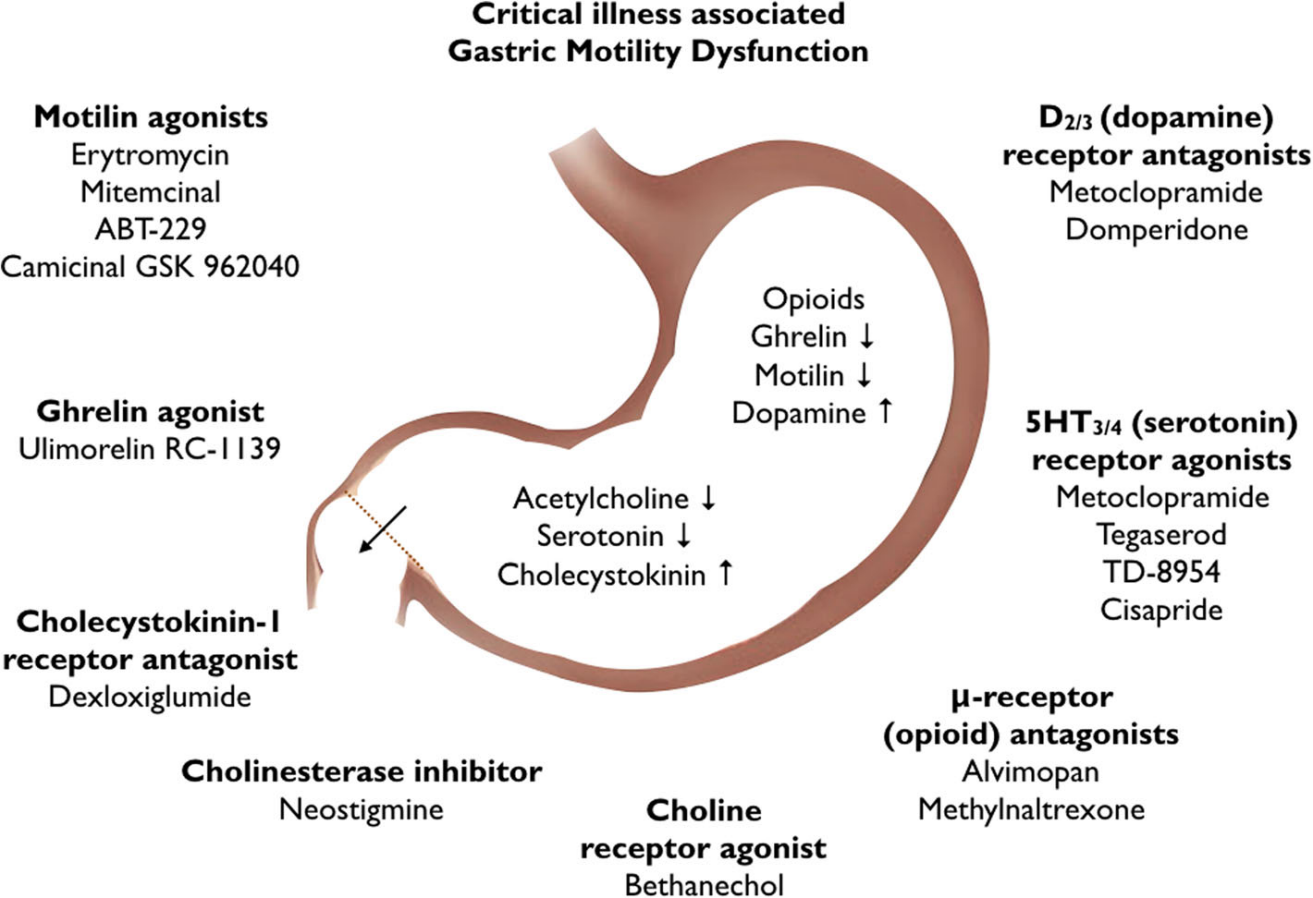
Potential pharmacological targets to treat critical illness-associated gastric motility dysfunction. During critical illness availability of acetylcholine to stimulate gastric smooth muscle is lower due to modulation of vagal tone and reduced levels of serotonin, motilin, and ghrelin resulting in reduced gastric emptying rate. Drugs that increase acetylcholine availability and receptor agonists of these (enteric) hormones may have potential prokinetic activity. Selective receptor blockers of opioids (in case of opioid use) and cholecystokinin or dopamine are other potential promotility agents

Critical illness-associated gastric motility dysfunction is multifactorial and predominantly comprises antral hypomotility, delayed gastric emptying, and reduced migrating motor complexes [12]. Gastric smooth muscle is activated by acetylcholine. In general, neurons that secrete acetylcholine are excitatory, enhance smooth muscle contraction, increase intestinal secretions, release enteric hormones, and dilate blood vessels. In a complex interplay of vagal tone and receptor activation or antagonism of insulin, glucagon, serotonin, dopamine, motilin, cholecystokinin, peptide YY, and ghrelin, among other factors, gastric emptying is regulated. During critical illness this regulation is disrupted and the gastric emptying rate is reduced. Several drug targets have been identified to develop new promotility agents (Fig. 1) and several new medications are under investigation [13].

In a recent systematic review and meta-analysis of 13 randomized controlled trials comparing prokinetics with placebo, Lewis and coworkers demonstrated that there is moderate-quality evidence that prokinetic agents reduce feeding intolerance in critically ill patients compared with placebo or no intervention [14]. They furthermore concluded that the impact on other clinical outcomes such as pneumonia, mortality, and ICU length of stay is unclear. Therefore, we need new highly effective prokinetics with limited or absent tachyphylaxis and no serious side effects.

Recently in *Critical Care* Chapman and coworkers reported that camicinal (GSK962040), a novel motilin agonist, at present only available for enteral administration, accelerates gastric emptying in feed-intolerant critically ill patients compared with placebo, resulting in augmented glucose absorption and improved gastric emptying [15]. Glucose absorption increased two-fold following administration of 50 mg of camicinal. This is important as the ultimate goal is not only to prevent complications of gastric feeding but, more importantly, to improve feeding performance reflected by a better absorption of nutrients.

Although the small sample size (15 camicinal patients versus eight placebo patients), no detectable plasma levels in two patients—an observation difficult to understand—and no comparison with the commonly used prokinetics metoclopramide and erythromycin are study limitations, results are promising as no safety issues were detected, such as QTc-interval prolongation. Due to the study design, information on tachyphylaxis is still lacking.

Several other studies in critically ill patients addressing the performance of novel prokinetics are ongoing or are soon to commence in international study sites (PROMOTE Trial studying the efficacy of the ghrelin agonist ulimorelin versus metoclopramide (NCT02784392) and the NUTRIATE Study comparing camicinal (GSK962040) 50 mg versus metoclopramide (NCT01934192).

Until robust study results in critically ill patients comparing new prokinetic agents with commonly used prokinetics have shown the safety and efficacy of these new promotility agents, we still have to rely on metoclopramide and erythromycin as prokinetics to improve feeding performance in patients at high risk for aspiration and critical illness-associated gastric motility dysfunction while closely monitoring side effects.

However, motility research is moving rapidly towards new therapeutic options to improve our pharmacological armamentarium for critical illness-associated gastric motility dysfunction.

## Abbreviations

ASPEN: American Society for Parenteral and Enteral Nutrition
EN: Enteral nutrition
GFI: Gastrointestinal feeding intolerance
GRV: Gastric residual volume
ICU: Intensive care unit
SCCM: Society of Critical Care Medicine

## References

[1] Reintam Blaser A, Jakob SM, Starkopf J (2016). Gastrointestinal failure in the ICU. Curr Opin Crit Care..

[2] Metheny NA, Stewart BJ, Mills AC (2012). Blind insertion of feeding tubes in intensive care units: a national survey. Am J Crit Care..

[3] Stechmiller JK, Treloar D, Allen N (1997). Gut dysfunction in critically ill patients: a review of the literature. Am J Crit Care..

[4] McClave SA, Taylor BE, Martindale RG (2016). Guidelines for the provision and assessment of nutrition support therapy in the adult critically ill patient: Society of Critical Care Medicine (SCCM) and American Society for Parenteral and Enteral Nutrition (A.S.P.E.N.). JPEN J Parenter Enteral Nutr.

[5] Poulard F, Dimet J, Martin-Lefevre L (2010). Impact of not measuring residual gastric volume in mechanically ventilated patients receiving early enteral feeding: a prospective before-after study. JPEN J Parenter Enteral Nutr..

[6] Reignier J, Mercier E, Le Gouge A (2013). Effect of not monitoring residual gastric volume on risk of ventilator-associated pneumonia in adults receiving mechanical ventilation and early enteral feeding: a randomized controlled trial. JAMA..

[7] McClave SA, DeMeo MT, DeLegge MH (2002). North American summit on aspiration in the critically ill patient: consensus statement. JPEN J Parenter Enteral Nutr..

[8] Heyland DK, Murch L, Cahill N (2013). Enhanced protein-energy provision via the enteral route feeding protocol in critically ill patients: results of a cluster randomized trial. Crit Care Med..

[9] Taylor SJ, Fettes SB, Jewkes C, Nelson RJ (1999). Prospective, randomized, controlled trial to determine the effect of early enhanced enteral nutrition on clinical outcome in mechanically ventilated patients suffering head injury. Crit Care Med..

[10] Grant K, Thomas R (2009). Prokinetic drugs in the intensive care unit: reviewing the evidence. JICS..

[11] Van der Meer YG, Venhuizen WA, Heyland DK, van Zanten AR (2014). Should we stop prescribing metoclopramide as a prokinetic drug in critically ill patients?. Crit Care..

[12] Ukleja A (2010). Altered GI, motility in critically Ill patients: current understanding of pathophysiology, clinical impact, and diagnostic approach. Nutr Clin Pract..

[13] Van Zanten AR, van der Meer YG, Venhuizen WA, Heyland DK (2015). Still a place for metoclopramide as a prokinetic drug in critically ill patients?. JPEN J Parenter Enteral Nutr..

[14] Lewis K, Alqahtani Z, Mcintyre L (2016). The efficacy and safety of prokinetic agents in critically ill patients receiving enteral nutrition: a systematic review and meta-analysis of randomized trials. Crit Care..

[15] Chapman MJ, Deane AM, O'Connor SL (2016). The effect of Camicinal (GSK962040), a motilin agonist, on gastric emptying and glucose absorption in feed-intolerant critically ill patients - a randomized, blinded, placebo-controlled, clinical trial. Crit Care.

